# ICTV Virus Taxonomy Profile: *Marnaviridae* 2021

**DOI:** 10.1099/jgv.0.001633

**Published:** 2021-08-06

**Authors:** Andrew S. Lang, Marli Vlok, Alexander I. Culley, Curtis A. Suttle, Yoshitake Takao, Yuji Tomaru, ICTV Report Consortium

**Affiliations:** ^1^​ Department of Biology, Memorial University of Newfoundland, St John’s, NL, Canada; ^2^​ Department of Biochemistry and Molecular Biology, University of British Columbia, Vancouver, BC, Canada; ^3^​ Institut de Biologie Intégrative et des Systèmes, Université Laval, Québec, QC, Canada; ^4^​ Departments of Botany, Earth, Ocean and Atmospheric Sciences and Microbiology and Immunology, Institute for the Oceans and Fisheries, University of British Columbia, Vancouver, BC, Canada; ^5^​ Department of Marine Bioscience, Fukui Prefectural University, Fukui, Japan; ^6^​ Fisheries Technology Institute, Japan Fisheries Research and Education Agency, Hatsukaichi, Hiroshima, Japan

**Keywords:** ICTV Report, *Marnaviridae*, taxonomy

## Abstract

The family *Marnaviridae* comprises small non-enveloped viruses with positive-sense RNA genomes of 8.6–9.6 kb. Isolates infect marine single-celled eukaryotes (protists) that come from diverse lineages. Some members are known from metagenomic studies of ocean virioplankton, with additional unclassified viruses described from metagenomic datasets derived from marine and freshwater environments. This is a summary of the International Committee on Taxonomy of Viruses (ICTV) Report on the family *Marnaviridae*, which is available at ictv.global/report/marnaviridae.

## Virion

**Fig. 1.**
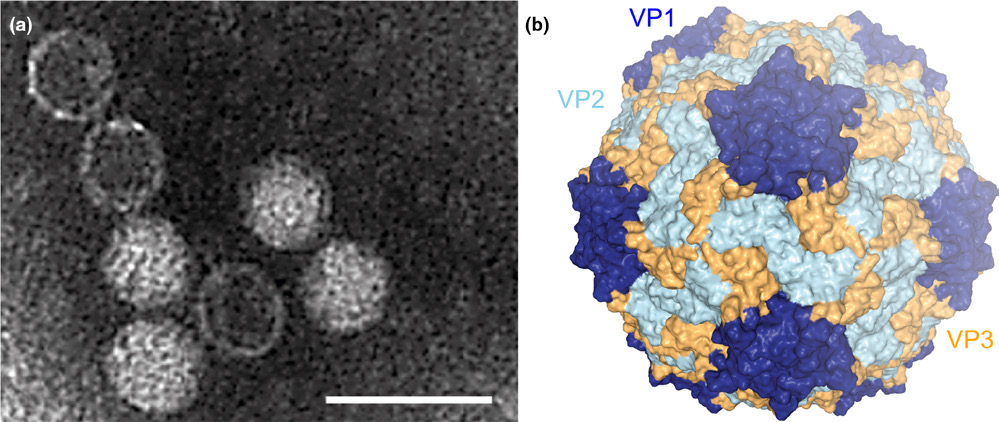
*Marnaviridae* virion structure. (a) Negatively stained transmission electron micrograph of Heterosigma akashiwo RNA virus virions (scale bar=50 nm). (b) Cryo-electron microscopy reconstructed capsid structure at 3.1 Å resolution of Chaetoceros tenuissimus RNA virus type II (species *Chaetenuissarnavirus II*, genus *Sogarnavirus*) (PDB structure 6SHL viewed in NGL-WebGL). The three major capsid proteins are represented in dark blue (VP1), light blue (VP2) and yellow (VP3).

Structurally characterized members have polyhedral virions, 22–35 nm in diameter, without envelopes or discernible projections (Table 1, Fig. 1). There are four conserved structural proteins, VP1–4. Capsid protein and virion structures have been resolved by cryo-electron microscopy for Chaetoceros tenuissimus RNA virus type II (species *Chaetenuissarnavirus II*) [1].

**Table 1.** Characteristics of members of the family *Marnaviridae*

Example:	Heterosigma akashiwo RNA virus (AY337486), species *Heterosigma akashiwo RNA virus*, genus *Marnavirus*
Virion	Non-enveloped, 22–35 nm with four structural proteins
Genome	8.6–9.6 kb of positive-sense, non-segmented RNA
Replication	Cytoplasmic, involving RNA-directed RNA polymerase and helicase; cytolytic
Translation	Directly from genomic RNA containing one or more internal ribosomal entry sites
Host range	Single-celled eukaryotes (protists) from marine environments
Taxonomy	Realm *Riboviria*, kingdom *Orthornavirae*, phylum *Pisuviricota*, class *Pisoniviricetes*, order *Picornavirales*; >5 genera and >15 species

## Genome

**Fig. 2.**
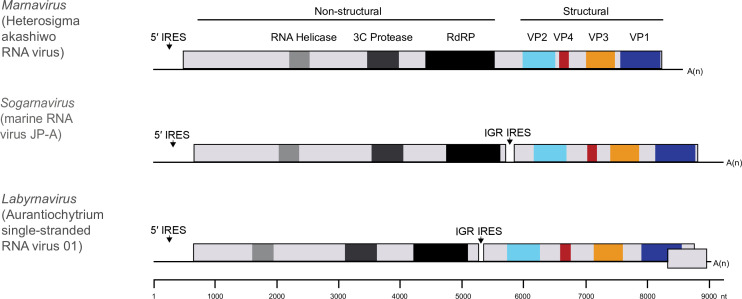
Genome architectures of viruses in the family *Marnaviridae*. IRES, internal ribosome entry site. RdRP, RNA-directed RNA polymerase. There is a putative IRES in the intergenic region (IGR) of the di-cistronic genomes.

Members possess monopartite positive-sense RNA genomes of 8.6–9.6 kb [2] containing one (*Locarnavirus*, *Marnavirus*) or two ORFs (*Locarnavirus*, *Kusarnavirus*, *Bacillarnavirus*, *Salisharnavirus*, *Sogarnavirus*); members of the genus *Labyrnavirus* have a third small overlapping open reading frame (ORF) at the 3′-end of the genome (Fig. 2). Virus genomes encode conserved helicase, RNA-directed RNA polymerase and structural protein domains. Some genomes also encode regions that resemble the 3C cysteine proteinases of members of the family *Picornaviridae*. Predicted secondary structures within the 5′-non-coding regions and intergenic regions suggest the presence of internal ribosome entry sites. A 3′-poly(A) tail terminates the genome of those viruses that have been isolated.

## Replication

Viral proteins are synthesized as part of one or two polyproteins. Regardless of whether the genomes are mono- or di-cistronic, the non-structural proteins are encoded in the 5′-region and the structural proteins are encoded in the 3′-region. Replication is cytolytic and various cytopathic effects have been observed [3–8]. Membrane vesicle structures associated with the endoplasmic reticulum [3] are likely to represent sites of RNA synthesis.

## Taxonomy

Current taxonomy: www.ictv.global/taxonomy. The genus *Marnavirus* includes the species *Heterosigma akashiwo RNA virus*, whose only member has a mono-cistronic genome and infects a harmful algal bloom-forming raphidophyte. *Labyrnavirus* includes the species *Aurantiochytrium single-stranded RNA virus 01*, whose only member has a di-cistronic genome and a third small overlapping ORF, which is transcribed as a sub-genomic RNA during infection but may not be functional [9]. The host is *Aurantiochytrium* sp., a marine thraustochytrid. *Locarnavirus* includes the species *Jericarnavirus B*, *Sanfarnavirus 1*, *Sanfarnavirus 2* and *Sanfarnavirus 3*, members of which were discovered through metagenomic analysis of marine virioplankton. Both mono- and di-cistronic genome organizations are found. *Kusarnavirus* includes the species *Astarnavirus*, whose only member infects the diatom *Asterionellopsis glacialis* and has a di-cistronic genome. *Bacillarnavirus* includes the species *Rhizosolenia setigera RNA virus 01*, *Chaetoceros tenuissimus RNA virus 01* and *Chaetoceros socialis forma radians RNA virus 1*, members of which infect diatoms and have di-cistronic genomes. *Salisharnavirus* includes the species *Britarnavirus 1*, *Britarnavirus 4*, *Palmarnavirus 128* and *Palmarnavirus 473*, members of which were discovered by metagenomics and have di-cistronic genomes. *Sogarnavirus* includes the species *Chaetenuissarnavirus II*, *Chaetarnavirus 2*, *Britarnavirus 2*, *Britarnavirus 3*, *Palmarnavirus 156* and *Jericarnavirus A*. Members have di-cistronic genomes and have been isolated from diatoms or discovered by metagenomics.

## Resources

Full ICTV Report on the family *Marnaviridae*: https://www.ictv.global/report/marnaviridae
